# Molecular Surveillance for Imported Antimicrobial Resistant *Plasmodium falciparum*, Ontario, Canada

**DOI:** 10.3201/eid2804.210533

**Published:** 2022-04

**Authors:** Ruwandi Kariyawasam, Rachel Lau, Eric Shao, Katherine Tan, Adrienne Showler, Filip Ralevski, Samir N. Patel, Andrea K. Boggild

**Affiliations:** University of Alberta, Edmonton, Alberta, Canada (R. Kariyawasam);; Alberta Precision Labs-Public Health, Edmonton (R. Kariyawasam);; Public Health Ontario Laboratory, Toronto, Ontario, Canada (R. Lau, F. Ralevski, S.N. Patel);; University of Western Ontario, London, Ontario (E. Shao);; University of Toronto, Toronto (K. Tan, A. Showler, S.N. Patel, A.K. Boggild);; Georgetown University, Washington, DC, USA (A. Showler);; Toronto General Hospital, Toronto (A. Showler, A.K. Boggild)

**Keywords:** malaria, antimicrobial resistance, *Plasmodium falciparum*, *Plasmodium*, vector-borne infections, parasites, imported infections, travel medicine, tropical medicine, Canada

## Abstract

Single-nucleotide polymorphisms at several loci have been correlated with *Plasmodium falciparum* drug resistance. We examined the prevalence of resistance markers in *P. falciparum* from imported malaria cases in Canada during 3 time periods, 2008–2009, 2013–2014, and 2017–2018. We evaluated single-nucleotide polymorphisms at *atpase6* (pfATPase6), *pfcrt* (chloroquine resistance transporter)*,* *cytb* (cytochrome b), *dhfr* (dihydrofolate reductase), *dhps* (dihydropteroate synthetase), *mdr1* (multidrug resistance protein) and *mdr1* copy number, and *kelch13* (kelch protein gene on chromosome 13). Over time, we observed increasing mutant genotypes for *dhfr* S108N and *dhps* A613T and decreasing mutant genotypes for *mdr1* N86Y, D1246Y, *pfcrt* K76T, and *pfcrt* 74–75; we identified no *kelch13* mutations. We observed fewer mutations indicative of chloroquine resistance over time, which may reflect reduced chloroquine pressure in specimens from travelers to Africa. Mutations conferring proguanil resistance increased over time. Minor genotypes confirm the heterogeneous nature of infection and may affect treatment success.

Malaria remains the deadliest vectorborne infectious disease worldwide (*1*). *Plasmodium* spp., most commonly *P. falciparum*, are responsible for ≈229 million cases and ≈500,000 deaths from malaria annually (*2*). Although malaria incidence and death have decreased over the past decade, emerging antimalarial drug resistance, fueled by counterfeiting, overuse, and underdosing, threatens control and elimination efforts (*2*). *P. falciparum* resistance mutations have resulted in waning efficacy in multiple antimalarial classes including artemisinins, quinolines, and antifolates (*3*). Increased international travel and climate change are exacerbating the spread of malarial vectors (*4*), making drug-resistant *P. falciparum* malaria imported from endemic regions, in particular Africa and Southeast Asia, a growing concern (*5*). Surveillance of antimalarial drug resistance, particularly among *P. falciparum* specimens, is crucial both to inform universal treatment guidelines and to identify global patterns of emerging resistance. We aimed to identify the prevalence of several resistance markers, including genes that confer resistance to chloroquine, mefloquine, atovaquone/proguanil, and artemisinins, and to quantify the copy number of multidrug resistance genes (*pfmdr1*) in *P. falciparum* isolates from malaria cases imported to Ontario, Canada, over a 10-year period. 

## Methods

### Specimens

From the malaria biobank at the Public Health Ontario Laboratory in Toronto, Ontario, Canada, we retrieved unique surplus whole-blood clinical specimens containing *P. falciparum* from the years 2008–2009, 2013–2014, and 2017–2018. We confirmed the *P. falciparum* specimens from the biobank as monoinfections using multiplex real-time quantitative PCR (qPCR) (*1*,*6*,*7*) after clinical testing, which included Giemsa-stained thick and thin blood film examination by certified medical lab technologists and rapid diagnostic test using the Abbott BinaxNOW malaria kit (https://www.globalpointofcare.abbott/en/index.html). To eliminate the possibility of recrudescent infections, we excluded specimens from patients who had positive blood smears in the year before our enrollment period.

### Extracting DNA from Whole Blood and Detecting Parasite DNA by Real-Time qPCR

We extracted DNA from surplus blood (0.4 mL) using the KingFisher Pure DNA Blood Kit (Thermo Fisher Scientific, https://www.thermofisher.com) and stored it in 50-μL aliquots: one at −20°C for working stock and another at −80°C for storage stock. We used included BEI Resources W2 MRA-157 (https://www.beiresources.org) and ATCC 3D7 (https://www.atcc.org) samples as *P. falciparum*–positive controls. We tested samples using 2 species-specific duplex real-time qPCRs (*P. falciparum* and *P. vivax*; *P. malariae* and *P. ovale*) (*6*,*7*). Samples were 25 μL total volume, including 12.5 μL Taqman Universal MasterMix (Thermo Fisher) and 5 μL template DNA. To perform all reactions, we used the Applied Biosystems 7500 Fast Real-Time PCR system (Thermo Fisher) for 2 min at 50°C and 10 min at 95°C, followed by 45 cycles of 15 s at 95°C and 1 min at 60°C.

### Pyrosequencing

We analyzed 23 different single-nucleotide polymorphisms (SNPs) across 6 genes: *atpase6* (PfATPase6; SNPs A623E, S769N), *cytb* (cytochrome b; SNP Y268NSC), *dhfr* (dihydrofolate reductase; SNPs A16V, C50R, N51I, C59R, S108N, I164L), *dhps* (dihydropteroate synthetase; SNPs S436FA, A437G, K540E, A581G, A613TS), *mdr1* (multidrug resistance protein; SNPs N86Y, Y184F, S1034CTR, N1042D, D1246Y), and *pfcrt* (chloroquine resistance transporter; SNPs K76T, N75E, M74I, C72S). We performed 16 PCR reactions according to the Pyromark PCR Kit protocol (QIAGEN, https://www.qiagen.com) using 400 nmol/L–concentration primers published elsewhere (Table 1). We performed all reactions on ABI Veriti Thermal Cyclers (Thermo Fisher) with adjusted annealing temperatures of 44°C for *dhfr* 164 and 63.8°C for *pfcrt*. We ran 3 μL of PCR product on a 1% agarose gel at 100 V for 30 min to ensure band presence before pyrosequencing.

**Table 1:** Primer sets used for pyrosequencing and Sanger sequencing for *Plasmodium falciparum* resistance testing*

Primer†	Sequence, 5′ → 3′
Pf ATPase 6 codon 623	
Forward primer	CACCAAGGGGTATCAACAAATTC
Biotinylated reverse primer (HPLC purified)	CTTGTGCCATGCTCAAATGTATT
Sequence primer	TCTAATTATACTACAGCTCA
Pf ATPase 6 codon 769	
Biotinylated forward primer (HPLC purified)	CAAAATATGGGAAAAAGAGCATTA
Reverse primer	TCTGGCCGTATTAATATTATCACC
Sequence primer	ATAAATTAAATCTTGTTCTA
Pf *cytb*	
Forward primer	CGTCCTGATAATGCTATCGTAGTAAATAC
Biotinylated reverse primer (HPLC purified)	CAGGCATCTAACATGGATATAGCA
Sequence primer	TGGTACTTTCTACCATT
*pfcrt*	
Biotinylated forward primer (HPLC purified)	GGAGGTTCTTGTCTTGGTAAA
Reverse primer	CAGGCATCTAACATGGATATAGCA
Sequence primer	TTTGTTTAAAGTTCTTTTAG
Pf MDR1 codon 86	
Forward primer	CGTTTAAATGTTTACCTGCACAA
Biotinylated reverse primer (HPLC purified)	TTGTCCATCTTGATAAAAAACACT
Sequence primer	GTGTAATATTAAAGAACATG
Pf MDR1 codon 184	
Forward primer	AGTTCAGGAATTGGTACGAAATTT
Biotinylated reverse primer (HPLC purified)	AACGTGCATTTTTTATTAATGACC
Sequence primer	CCAGTTCCTTTTTAGGTT
Pf MDR1 codon 1034, 1042	
Forward primer	GCGGAGTTTTTGCATTTAGTTCA
Biotinylated reverse primer (HPLC purified)	TTAAGAAGGATCCAAACCAATAGG
Sequence primer	GCAGCTTTATGGGGAT
Pf MDR1 codon 1246	
Biotinylated forward primer (HPLC purified)	TTTTCAAACCAATCTGGATCTG
Reverse primer	CGTTTAACATCTTCCAATGTTGC
Sequence primer	ATTGAAAATAAGTTTCTAAG
Pf *dhfr* codon 16	
Biotinylated forward primer	AACAAGTCTGCGACGTTTTCGATA
Reverse primer	CCCCTCATTTTTGCTTTCAACC
Sequence primer	GCTTTCAACCTTACAACA
Pf *dhfr* codon 51, 51, 59	
Forward primer	TGAGGGGAAAAAAAATGAGGTT
Biotinylated reverse primer (HPLC purified)	AAGGTTTAAATTTTTTTGGAATGC
Sequence primer	AAAGGAGTATTACCATGGA
Pf *dhfr* codon 108	
Biotinylated forward primer	CAAAATGTTGTAGTTATGGGAAGA
Reverse primer	TTTAAATTTTTTTGGAATGCTTTC
Sequence primer	TGGAATGCTTTCCCAG
Pf *dhfr* codon 164	
Biotinylated forward primer (HPLC purified)	GTTTTACTTGGGAAATTAAATTACT
Reverse primer	TCTGGAAAAAATACATCACATTC
Sequence primer	ACAACGGAACCTCCTA
Pf *dhps* codon 436, 437	
Forward primer	ACGTGCTGTTCAAAGAATGTTT
Biotinylated reverse primer (HPLC purified)	GTTTCGCATCACATTTAACAATT
Sequence primer	ATAGATATAGGTGGAGAATC
Pf *dhps* Codon 540	
Biotinylated forward primer (HPLC purified)	GCATAAAAGAGGAAATCCACATAC
Reverse primer	CGAGGTATTCCATTTAATACAAGA
Sequence primer	ATTATCATAATTTGTTAGTT
Pf *dhps* codon 581	
Forward primer	ACCTCGTTATAGGATACTATTTGA
Biotinylated reverse primer (HPLC purified)	TCCAATAAAAAGTGGATACTCATC
Sequence primer	TTGATATTGGATTAGGATTT
Pf *dhps* codon 613	
Biotinylated forward primer (HPLC purified)	TCCACTTTTTATTGGATATTCAAG
Reverse primer	CTGAAACATCCAATTGTGTGAT
Sequence primer	CATTCATGCAATGGG
*kelch13*	
K13_PCR_F	CGGAGTGACCAAATCTGGA
K13_PCR_R	GGGAATCTGGTGGTAACAGC
K13_N1_F	GCAAGCTGCCATTCATTTG
K13_N1_R	GCCTTGTTGAAAGAAGCAGA
K13_N2_F	CGCCAGCATTGTTGACTAAT
K13_N2_R	GCGGAGTAGTAGCGAGAAT

*Pyrosequencing using Pyromark PCR Kit protocol (QIAGEN, https://www.qiagen.com). *cytb*, cytochrome b; *dhfr*, dihydrofolate reductase; *dhps*, dihydropteroate synthetase; HPLC, high performance liquid chromatography; MDR, multidrug resistance; Pf, *P. falciparum*; *pfcrt*, *P. falciparum* chloroquine resistance transporter †See (*10*).

We performed pyrosequencing using the Pyromark Q24 Vacuum Workstation and Pyromark Q24 Pyrosequencer (QIAGEN) according to manufacturer protocols. We designed 21 pyrosequencing assays using PSQ assay design software version 1.0.6 (https://psq-assay-design.software.informer.com) (Table 1). To determine run-to-run consistency and reproducibility, we calculated the intra-assay coefficient of variation by running the *cytb* assay on 3 samples in quintuplicate. We analyzed samples using Pyromark Q24 version 1.0.10 software (QIAGEN).

### Sanger Sequencing

We interrogated *kelch13* (kelch protein gene on chromosome 13) for 20 SNPs according to Institut Pasteur protocols using primers published elsewhere (*8*,*9*) (Table 1). To validate the results obtained from pyrosequencing, we performed a quality control check of the gene targets using the first 10% of cases from each time period and controls. We designed sequencing primers with Primer 3 (*10*,*11*) and performed PCR using primers published elsewhere (*8*,*9*,*12*) that target drug resistance genes according to the Accuprime Pfx SuperMix protocol (Thermo Fisher) (Table 1); we adjusted the annealing temperature to 60°C and cycles to 40 for *cytb* and 45 for all *mdr1* and *dhfr* assays. We ran 3 μL of PCR product on a 1% agarose gel at 100 V for 30 min to ensure band presence before sequencing.

We performed sequencing reactions using the Big Dye Terminator v3.1 Cycle Sequencing Kit (Thermo Fisher) with a volume of 20 μL, including 1 μL of PCR product, 2 μL of Big Dye ready reaction mix, 3 μL of Thermo Fisher sequencing buffer, and 2 μL of 10 μmol/L of primer. We used a Bio-Rad C1000 Thermal Cycler (https://www.bio-rad.com) for 1 min at 96°C, 25 cycles of 10 s at 96°C, 5 s at 50°C, and 4 min at 60°C. We purified product using an in-house isopropanol protocol including incubating at room temperature using 15 μL of 100% isopropanol for 15 min, centrifuging at 3,000 × *g* for 30 min at room temperature, centrifuging at 2,000 × *g* for 10 min after 35 μL of 70% isopropanol had been added, then inverting the plate and centrifuging for 700 × *g* at room temperature for 1 min. Last, we added 20 μL of Hi-Di Formamide (Thermo Fisher) to reconstitute each sample and sequenced using an Applied Biosystems 3730xl DNA Analyzer (Thermo Fisher). We standardized data using a Thermo Fisher sequencing analysis program and used a BioEdit sequence alignment editor version 7.2.5 (https://bioedit.software.informer.com) to analyze the sequence for nucleotide changes at each identified SNP location.

### *Pfmdr1* Copy Number Analysis

We tested specimens for *pfmdr1* copy number using real-time qPCR with primers and probes published elsewhere (*12*), using a total volume of 25 μL, including 12.5 μL Taqman Universal MasterMix (Thermo Fisher) and 5 μL template DNA. We performed reactions on an Applied Biosystems 7500 Fast Real-Time PCR system (Thermo Fisher) using the same conditions used to detect parasite DNA. We ran the process in triplicate for specimens and used the 2^-ΔΔCT^ method to calculate relative expression (*13*).

### Statistical Analyses

We considered a genotype mutant dominant if the mutant allelic frequency within the specimen was >50% and wildtype dominant if <50%. We calculated median and range *pfmdr1* relative copy numbers for each time period and compared all categorical variables (proportions of mutant versus wildtype genotypes) using the Fisher exact test. We calculated median and range for mutant allelic frequencies within specimens and presented them descriptively across time periods to inform surveillance for resistance mutations over time. We compared differences in median *pfmdr1* relative copy numbers across time periods using the Kruskal-Wallis H test and set significance at p <0.05. We analyzed data using Stata version 13 software (StataCorp LLC, https://www.stata.com).

## Results

Over the enrollment period, we retrieved a total of 574 primary blood specimens from the malaria biobank containing >1 *Plasmodium* spp. determined using real-time qPCR; 566 (98.6%) specimens contained just 1 species of *Plasmodium* and the other 8 (1.4%) contained 2 *Plasmodium* species. Of specimens with single-species infections, 365/574 (64%) were positive only for *P. falciparum*, 142/574 (25%) only for *P. vivax*, 46/574 (8%) only for *P. ovale*, and 13/574 (2.3%) only for *P. malariae*. Of the specimens with 2 *Plasmodium* species, 3/574 (0.5%) were *P. falciparum–P. ovale* co-infections and 5/574 (0.9%) were *P. ovale–P. vivax* co-infections. Only 243/365 (67%) specimens with *P. falciparum* monoinfection were identified as unique cases, 75 (31%) occurring during July 2008–June 2009, 79 (33%) during July 2013–June 2014, and 89 (37%) during July 2017–June 2018. Of those 243 *P. falciparum*–only specimens from unique cases, 169 (70%) were from male patients, 66 (27%) from female patients, and 8 (3%) had no reported sex; there was no difference in proportions of sex across time periods (p = 0.47) (Table 2). Mean age was 39.2 (range 3–88) years, which did not differ between time periods (p = 0.61) (Table 2). Mean parasitemia was 0.3% (<0.01%–24.0%) across all 3 time periods, and we saw no significant differences over time (p = 0.10) (Table 2). A total of 186 (77%) patients had documented travel history, 81 (33%) in West Africa, the most common region, and 40 (16%) in Nigeria, the most common country (Table 2). Only 5/243 (2%) unique *P. falciparum* specimens were from travelers to Southeast Asia, and 1 (0.4%) was from a traveler to the Caribbean (Table 2). 

**Table 2:** Traveler characteristics by time period in which *P. falciparum* infection was detected, Ontario, Canada*

Characteristic	Total, N = 243	2008–2009, n = 75	2013–2014, n = 79	2017–2018, n = 89	p value
Age, y, mean (SD) †	39.2 (18.3)	40.9 (17.1)	38.0 (16.6)	38.9 (20.6)	0.61
Sex‡					0.47
F	66 (27.1)	18 (24.0)	19 (24.0)	29 (32.6)	
M	169 (69.5)	53 (70.7)	57 (72.2)	59 (66.3)	
Unknown	8 (3.3)	4 (5.3)	3 (3.8)	1 (1.1)	
Parasitemia, %, median, (range)§	0.3 (<0.1–24.0)	0.3 (<0.1–17.8)	0.3 (<0.1–12.0)	0.7 (<0.1–24.0)	0.1
Region of acquisition, no. (%)¶					0.14
West Africa	81 (33.3)	20 (26.7)	17 (21.5)	38 (42.7)	
Nigeria#	40 (16.5)	10 (13.3)	8 (10.1)	22 (24.7)	
East Africa	18 (7.4)	4 (5.3)	8 (10.1)	6 (6.7)	
Sudan**	7 (2.9)	1 (0.25)	5 (6.3)	1 (1.1)	
Africa, other	3 (1.2)	1 (1.3)	1 (1.3)	1 (1.1)	
Africa, not otherwise specified	30 (12.3)	11 (14.7)	12 (15.2)	7 (7.9)	
Caribbean, Dominican Republic	1 (0.4)	1 (1.3)	0	0	
Southeast Asia	5 (2.1)	0	4 (5.1)	1 (1.1)	
South America: Guyana	1 (0.4)	0	0	1 (1.1)	
Unknown	104 (42.8)	33 (44)	36 (45.6)	35 (39.3)	

*Values are no. (%) except as indicated. †Age was missing for 4 patients in 2008–2009, 1 patient in 2013–2014, and 1 patient in 2017–2018. ‡Sex was missing for 4 patients in 2008–2009, 3 patients in 2013–2014, and 1 patient in 2017–2018. §Parasitemia was missing for 2 patients in 2008–2009. ¶West Africa: Congo, Burkina Faso, Cameroon, Ghana, Guinea, Ivory Coast, Liberia, Nigeria, Togo, Senegal, and Sierra Leone; East Africa: Burundi, Ethiopia, Kenya, Rwanda, Sudan, South Sudan, Tanzania and Uganda; Africa, other: Zambia, Libya, and South Africa; Africa not otherwise specified: travel to multiple regions in Africa, or specific destination within Africa not reported. #Top West Africa source country. **Top East Africa source country.

### Prevalence of Mutations in Mutant Dominant Genotypes

All 243 unique *P. falciparum­–*only specimens contained >1 resistance mutation. We detected the highest prevalence of mutant genotypes in *dhfr*; 212 (91%) cases demonstrated triple codon mutation N51I, C59R, and S108N (Appendix Table 1). *dhfr* SNP analysis revealed an increase in S108N mutant genotypes from 89% in 2008–2009 to 100% in 2017–2018 (Appendix Table 1). *pfcrt* SNP analysis revealed a gradual decline in K76T mutant genotype from 57% in 2008–2009 to 33% in 2017–2018 (p = 0.011), coupled with a decrease in M74I and N75E from 52% in 2008–2009 to 27% in 2017–2018 (p = 0.010; Appendix Table 2). *dhps* A613T SNP analysis revealed an overall increase in mutant genotypes, from 12% in 2008–2009 to 28% in 2017–2018 (p = 0.024; Appendix Table 3). *mdr1* N86Y and D1246Y analysis revealed a decline in mutant genotypes from 2008–2009 and 2017–2018 (p<0.003; Appendix Table 4). We observed no difference in mutant genotype populations across time periods for *dhps* K540E, A581G, A437G, S436A, S436F *mdr1* Y184F, S1034T, or *atpase6* A623E (Appendix Tables 3–5). Last, we observed no mutant genotype populations for *cytb* Y268NSC across time periods (Appendix Table 6).

### Mutant Allelic Frequency within Wildtype Dominant Specimens

We presented summaries of mutant allelic frequencies within wild-type–dominant specimens (i.e., with <50% within-specimen mutation prevalence) for *dhfr* (Appendix Table 1), *pfcrt* (Appendix Table 2), *dhps* (Appendix Table 3), *mdr1* and *mdr1* copy number (Appendix Table 4), *atpase6* (Appendix Table 5), and *cytb* (Appendix Table 6). Many minor changes in allelic frequencies of questionable importance occurred across time periods (Appendix Tables 1–6). Notable findings include that *pfcrt* K76T and C72S mutant allele populations among wild-type infections declined significantly from 2008–2009 to 2017–2018 (Appendix Table 2). Conversely, mutant allele frequency within *mdr1* D1246Y increased from 7% in 2008–2009 to 16% in 2017–2018, despite a decline in overall proportion of mutant-dominant genotypes (i.e., with >50% within-specimen mutation frequency) from 17.6% to 3.5% across time periods (Appendix Table 4). Frequency of any allelic mutant among wildtype infections at *cytb* Y268N increased from 0% in 2008–09 and 2013–2014 to 3% in 2017–2018 (Appendix Table 6).

### *mdr1* Copy Number and *kelch13* Mutations

Using the 3D7 comparator reference strain, which has a *pfmdr1* copy number of 1, we found an increase in median *pfmdr1* copy number from 1.1 (range 0.83–1.4) in 2008–2009 to 1.9 (range 0.73–5.4) in 2017–2018 (p<0.001) (Appendix Table 4). *kelch13* SNP analysis of >20 codons revealed no mutations for all analyzed specimens, despite 5 specimens with confirmed *P. falciparum* being from Southeast Asia (data not shown). We observed 2 silent mutations at codon 553 and 561 in the 2017–2018 specimens (sequences deposited to GenBank under accession nos. OM489472 and OM489473); however, travel history was not recorded for those patients. 

## Discussion

We analyzed 243 unique cases of *P. falciparum* imported to Canada using pyrosequencing assays based on previously reported genetic markers known to confer drug resistance. Our results have provided stakeholders with a resource for tracking antimalarial resistance over time. Conventional SNP analysis involves Sanger sequencing and PCR–restriction fragment-length polymorphism, but over the past decade pyrosequencing has added utility for detecting drug-resistance markers (*14*). Now, pyrosequencing has been partially replaced by more powerful techniques, such as targeted next-generation sequencing, which remain accessible mostly just in reference or research laboratories because of bioinformatics requirements. Our analysis of *P. falciparum* markers of genotypic resistance using pyrosequencing provides evidence of a quantitative advantage over Sanger sequencing because allelic frequencies within wild-type populations can be monitored over time to capture emerging molecular resistance. We observed significant decreases in mutant genotypes for chloroquine resistance genes and an increase over time in the proportion of mutant genotypes for *dhfr*, the gene conferring resistance to proguanil. We also observed significant increases in markers of antifolate drug resistance correlating with increases in mutant allelic frequencies within wild-type populations.

The emergence of antimicrobial-resistant *P. falciparum* began in 1957 when the first cases of resistance to chloroquine were observed along the Cambodia–Thailand border (*15*). *pfcrt* encodes for a transmembrane protein in the digestive vacuoles of *P. falciparum* parasites. Mutations of the *pfcrt* gene, specifically at positions 72, 74, 75, and 76, confer resistance to chloroquine (*16*–*18*). *pfmdr1* mutation N86Y combined with increased *pfmdr1* copy numbers, both of which have been documented in many parts of Southeast Asia and recently in sub-Saharan Africa, is also implicated in chloroquine resistance (*12*,*17*,*19*–*21*). Our data document a decline over time in *pfcrt* mutants from positions 72 and 76 in imported cases of *P. falciparum*, indicating molecular reversion in chloroquine resistance at these codons. This phenomenon may reflect the reduction in chloroquine drug pressure on specimens from West Africa across the 10-year study period, during which oral artemisinin combination therapy has supplanted chloroquine use.

Drugs targeting folate metabolism, including pyrimethamine, sulfadoxine, and proguanil, are common components of fixed-dose combination antimalarials and inhibit purine and pyrimidine biosynthetic pathways (*3*). Point mutations in the *dhfr* and *dhps* genes conferring sulfadoxine and pyrimethamine resistance were identified as early as 1988 in Kenya. *dhps* mutations S436AF, A437G, A581G, and A613TS have been identified in sulfonamide-resistant specimens primarily found in Southeast Asia (*21*–*23*). Higher levels of resistance have been identified in specimens carrying multiple mutations conferring a synergistic effect, especially double mutation S436FA and A613TS (*23*). Overall, we documented an increase in all mutant *dhps* genotypes except K540E. Increases in mutant genotypes coincided with overuse of chloroquine alternatives, which have been heavily deployed in sub-Saharan Africa.

*dhfr* S108N is the essential driving force associated with resistance to pyrimethamine (*3*,*24*). Our data highlight evidence of antifolate resistance over time, which may relate to increasing reliance on antifolate drugs in the face of chloroquine resistance. Additional mutations including *dhfr* N51I, C59R, and I164 have been associated with more pyrimethamine-resistant specimens (*3*,*24*,*25*). We did not identify increases in either N51I or C59R mutations and detected only one I164L specimen in the 2008–2009 time frame, with no subsequent occurrences. 

Our findings are limited by a small sample set and incomplete travel history, but this limitation may suggest a decreased prevalence of I164L mutation in sub-Saharan Africa. Of interest, the *dhfr* A16V mutation generally occurs in conjunction with S108N, which is known to cause cycloguanil resistance (*26*). However, our data demonstrate no such correlation in the specimens we analyzed. Analyses by many groups over the years have led to the conclusion that greater numbers of point mutations in *dhfr* confer greater resistance, and cross-resistance between pyrimethamine and cycloguanil is a possibility, whereas S108N is the necessary first mutation responsible for pyrimethamine resistance (*3*). Our results coincide with previous literature resulting in data on a wave of antifolate resistance markers in specimens of *P. falciparum* imported to Ontario.

Artemisinin derivatives, the most powerful antimalarials used clinically, have been the main focus of concern surrounding recent efforts to mitigate *P. falciparum* antimicrobial resistance. After the initial surge of chloroquine resistance, which was quickly followed by a diminution of antifolate effectiveness, the World Health Organization recommended use of artemisinin-based combination therapies as first-line drugs of choice. However, similar to the experience when generations of antimalarial drugs have been heavily used in other endemic areas, many countries in Africa have seen strains of multi­drug-resistant *P. falciparum* appeared within 1 year of introducing artemisinin-based combination therapy formulations, occurring as early as 2003 (*27*). In Southeast Asia, artemisinin resistance has been associated with mutations in the propeller region of *P. falciparum* *kelch13* (*28*). At present, there is no documented large-scale spread of *kelch13* mutations in Africa; however, *kelch13* mutations have been documented in specimens from Bangladesh, suggesting a westward migration of mutations from their origins in the Thailand–Myanmar region (*29*–*35*).

Recently, there have been reports of *kelch13*-independent treatment failure with artemether/lumefantrine in *P. falciparum* cases imported to the United Kingdom by persons with travel history to Angola, Liberia, and Uganda (*30*). Despite novel mutations, including G112E, outside of the propeller-encoding domain in some patients, no reduced artemisinin susceptibility has been reported in the Greater Mekong region of Southeast Asia (*8*,*9*,*28*,*30*,*34*). Our data from specimens predominantly from West Africa correlate with these and other findings corroborating an absence of detectable mutations in 20 SNPs identified in the *kelch13* gene between codon 440 and the 3′ end of the coding region. On the other hand, *pfATP6*, which is also known as *pfSERCA* or *pfATPase6*, is a calcium ATPase gene involved in calcium ion transport. Point mutations at A623E and S769N in *pfATP6* could be associated with artemisinin resistance. Increased prevalence of these point mutations may be responsible for the rise of resistance in areas such as Cambodia, French Guiana, and Senegal, where artemisinin use is uncontrolled (*27*). Our data suggest that artemisinin-resistant *P. falciparum* infection is unlikely to occur in travelers returning to Canada, given that only 1 *atpase6* A623E mutant was detected in both the 2008–2009 and 2013–2014 time frames, and none for the *atpase6* S769N and *kelch13* mutations.

The fixed-dose combination atovaquone/proguanil, an antimalarial prophylactic popular among travelers, has been recommended more broadly in response to potential resistance to other antimicrobial classes, including artemisinins (*36*). However, documented cases with *cytb* mutations have resulted in treatment failure associated with resistance to atovaquone. We documented an exceptionally high prevalence of *P. falciparum* specimens (83%) with triple codon *dhfr* 51, 59, 108 mutation, which confer resistance to proguanil, leaving the partner drug, atovaquone, as the sole antimalarial effective in clearing *P. falciparum* (4 of every 5 cases in our setting). Any resistance to atovaquone, conferred by *cytb268* mutations, or impaired atovaquone absorption can lead to treatment failure (*37*,*38*); resistance as a result of *cytb* Y268NSC has been identified in travelers returning from Kenya, Angola, and many parts of East Africa (*22*,*39*–*41*). We observed no *cytb*-dominant mutants in our study, but in some specimens, the mutant allele frequency in the wild-type specimens were close to 20%, suggesting ≈1/5 of the infection could be comprised of mutant strains. Whether having a minor population of allelic mutants instead of dominant mutant allelic frequency equating to an overall mutant genotype can lead to treatment failure or recrudescence is unknown, but nevertheless, that condition may be an early indicator of emerging atovaquone resistance. Identifying subpopulations of resistant mutant strains can help determine appropriate treatment strategies and follow-up; prevalence of resistant strains as low as 10% can double in vitro 50% inhibitory concentration to chloroquine (*42*). This possibility reiterates the value of pyrosequencing and targeted next generation sequencing, which enable quantification of minor allelic populations.

Our conclusions may be limited by lack of complete data on travel history, history of malaria infection, and treatment strategies and outcomes among study participants. Subtle changes in mutant allelic frequency noted across time periods may be attributable to travel proclivities, period-to-period natural variation in expression, or a true change in prevalence. The technical limitations of developing a pyrosequencing assay targeting positions 74 and 75 only enabled qualitative analysis for *pfcrt* double mutations at those positions. A technical limitation of pyrosequencing includes diminished accuracy of determining proportions <5%. Although *kelch13* remains the only gene target analyzed via Sanger sequencing, minor alleles in mixed populations cannot be detected by Sanger sequencing; thus, pyrosequencing is a more useful and reliable sequencing technology for SNP analysis. Although pyrosequencing has largely been replaced by more effective techniques, such as targeted next-generation sequencing and possibly whole-genome sequencing, those newer techniques are limited by cost and the requirement for bioinformatics infrastructure and expertise, which generally confine their application to large reference or research laboratories. Future studies involving prospective recruitment of *P. falciparum*–infected travelers, with complete demographic, clinical, and treatment outcome data and use of targeted next-generation sequencing to identify other SNPs, risk factors, or confounders of resistance, could fill these knowledge gaps.

Surveillance of SNPs can serve as a sentinel of clinical resistance. Triple codon *dhfr* mutants, for example, were detected years before observation of increased sulfadoxine/pyrimethamine treatment failures in Kenya (*43*). Robust surveillance analyses over time of known markers conferring drug resistance in *P. falciparum* imported by travelers are lacking. We observed a high percentage of molecular resistance to proguanil and none to atovaquone. We observed an increase in imported antifolate-resistant of *P. falciparum* strains, including a decrease in chloroquine-resistant strains and virtually no signs of artemisinin resistance because of mutations at *kelch13.* Importation of resistant *P. falciparum* mono-infections highlights the importance of developing better surveillance tools to monitor drug resistance patterns based on time and source region. Such surveillance would also inform clinical decision-making and serve as a resource for tracking antimalarial resistance that clinicians could monitor for possible genetic markers of reduced drug efficacy.

Appendix Additional information on molecular surveillance for imported antimicrobial resistant *Plasmodium falciparum*
