# Supplementary material for: Molecular Surveillance for Imported Antimicrobial Resistant Plasmodium falciparum, Ontario, Canada
Source: Emerg Infect Dis. 2022 Apr;28(4):812–9. doi: 10.3201/eid2804.210533 (PMC8962893; doi:10.3201/eid2804.210533)
Supplement: Appendix — Additional information on molecular surveillance for imported antimicrobial resistant Plasmodium falciparum [file 21-0533-Techapp-s1.pdf]

Article DOI: https://doi.org/10.3201/eid2804.210533

Molecular Surveillance for Imported Antimicrobial Resistant *Plasmodium falciparum*, Ontario, Canada

Appendix

Appendix Table 1. Resistance mutations across *dhfr**

dhfr resistance mutations	All years (N = 243)	Time period 2008–2009 (N = 75)	2013–2014 (N = 79)	2017–2018 (N = 89)	P value
A16V†					
Mutant genotype, N (%)	0 (0%)	0 (0%)	0 (0%)	0 (0%)	1.0
Median frequency of MT allele among WT, % (range)	0.7 (0–19.4)	0.7 (0.3–4.7)	0.8 (0–19.4)	0.7 (0.5–2.4)	NA
S108N‡					
Mutant genotype, N (%)	227 (95.8%)	67 (89.3%)	74 (97.3%)	86 (100%)	0.001
Median frequency of MT allele among WT, % (range)	10.9 (0–45.5)	6.5 (0–45.5)	21.2 (15.0–27.5)	NA	NA
Median frequency of MT allele among MT, % (range)	99.1 (65.1–100)	98.9 (65.1–100)	98.3 (71.3–99.5)	99.5 (97.4–100)	NA
I164L§ (ATA → CTA or TTA)					
Median frequency of MT TTA allele, % (range)	0 (0–94.4)	0 (0–94.4)	0 (0–7.3)	0 (0–43.5)	NA
Median frequency of MT CTA allele, % (range)	0.1 (0–8.9)	0.15 (0–8.9)	0 (0–3.8)	0.7 (0–7.8)	NA
Median frequency of any mutation, % (range)	0.75 (0–96.8)	0.58 (0–96.8)	0 (0–11.1)	1.2 (0–43.5)	NA
Mutant genotype, N (%)	1 (0.4%)	1 (1.4%)	0 (0%)	0 (0%)	0.322
C50R¶					
Mutant genotype, N (%)	1 (0.4%)	0 (0%)	0 (0%)	1 (1.3%)	1.000
Median frequency of MT allele among WT, % (range)	0.9 (0–5.4)	0.8 (0–3.4)	0.9 (0–5.4)	0.8 (0–3.2)	NA
Median frequency of MT allele among MT, % (range)	97.2 (97.2–97.2)	NA	NA	97.2 (97.2–97.2)	NA
N51I**					
Mutant genotype, N (%)	214 (91.1%)	66 (88.0%)	72 (92.3%)	76 (92.7%)	0.553
Median frequency of MT allele among WT, % (range)	6.0 (0–48.1)	5.4 (2.1–48.1)	18.7 (0–39.8)	3.0 (0–7.4)	NA
Median frequency of MT allele among MT, % (range)	100 (57.8–100)	100 (60.1–100)	100 (61.8–100)	100 (57.8–100)	NA
C59R††					
Mutant genotype, N (%)	212 (93.0)	68 (90.7%)	74 (94.9%)	70 (93.3%)	0.583
Median frequency of MT allele among WT, % (range)	26.5 (0.8–49.9)	29.8 (16.5–49.9)	20.7 (0.8–25.6)	26.2 (24.2–45.8)	NA
Median frequency of MT allele among MT, % (range)	100 (50.4–100)	100 (63.4–100)	100 (50.4–100)	100 (55.2–100)	NA

*MT, mitochondrial; NA, not applicable WT, wild type.
†Missing: 2 (2013–2014); 24 (2017–2018)
‡Missing: 3 (2013–2014); 3 (2017–2018)
§Missing: 1 (2008–2009); 1 (2013–2014); 11 (2017–2018)
¶Missing: 1 (2013–2014); 10 (2017–2018)
**Missing: 1 (2013–2014); 7 (2017–2018)
††Missing: 1 (2013–2014); 14 (2017–2018)

Appendix Table 2. Resistance mutations across *pfcrt**

dfcrt resistance mutations	All years (N = 243)	Time period 2008–2009 (N = 75)	2013–2014 (N = 79)	2017–2018 (N = 89)	P value
K76T†					
Mutant genotype, N (%)	88 (43.6%)	42 (56.8%)	28 (38.4%)	18 (32.8%)	0.013
Median frequency of MT allele among WT, % (range)	11.4 (1.4–47.3)	15.9 (1.9–47.3)	13.5 (1.4–44.9)	5.8 (1.9–32.8)	NA
Median frequency of MT allele among MT, % (range)	97.2 (50.1–100)	97.5 (50.1–100)	97.6 (81.4–99.9)	95.9 (54.3–100)	NA
74-75‡					
Mutant genotype, N (%)	81 (39.9%)	39 (52%)	27 (37.5%)	15 (26.8%)	0.014
C72S§ (TGT→TCT)					
Mutant genotype, N (%)	0 (0%)	0 (0%)	0 (0%)	0 (0%)	1.000
Median frequency of MT allele among WT, % (range)	21.5 (3.1–39.1)	21.2 (3.1–39.1)	23.0 (3.4–36.8)	14.9 (9.3–23.6)	NA
C72S¶ (TGT→AGT)					NA
Mutant genotype, N (%)	2 (1.2%)	1 (1.4%)	1 (1.3%)	0 (0%)	NA
Median frequency of MT allele among WT, % (range)	28.6 (8.1–48.8)	29.6 (9.2–48.8)	28.7 (8.1–48.8)	16.0 (11.8–30.0)	NA
Median frequency of MT allele among MT, % (range)	73.4 (54.7–92.2)	54.7 (54.7–54.7)	92.2 (92.2–92.2)	NA	NA

*MT, mitochondrial; NA, not applicable WT, wild type.
†Missing: 6 (2008–2009); 1 (2013–2014); 34 (2017–2018)
‡Missing: 7 (2013–2014); 33 (2017–2018)
§Missing: 1 (2008–2009); 4 (2013–2014); 76 (2017–2018)
¶Missing: 1 (2008–2009); 4 (2013–2014); 76 (2017–2018)

Appendix Table 3. Resistance mutations across *dhps**

dhps resistance mutations	All years (N = 243)	Time period 2008–2009 (N = 75)	2013–2014 (N = 79)	2017–2018 (N = 89)	P value
K540E†					
Mutant genotype, N (%)	42 (17.4%)	10 (13.3%)	16 (20.5%)	16 (18.0%)	0.504
Median frequency of MT allele among WT, % (range)	0.6 (0.2–16)	0.5 (0.2–13.6)	2.4 (0.3–16)	0.5 (0.2–8.9)	NA
Median frequency of MT allele among MT, % (range)	95.2 (65.5–96.9)	95.5 (84.7–96.9)	95.3 (65.5–96.7)	94.5 (68.2–96.1)	NA
A581G‡					
Mutant genotype, N (%)	22 (10.0%)	7 (9.5%)	6 (8.0%)	9 (12.5%)	0.637
Median frequency of MT allele among WT, % (range)	0.9 (0–45.8)	0.1 (0–6.3)	2.0 (0–45.8)	0.7 (0–43.5)	NA
Median frequency of MT allele among MT, % (range)	94.4 (56.4–97.7)	92.8 (65.9–97.5)	92.2 (56.4–97.7)	96.3 (63.2–97.5)	NA
A613T§					
Mutant genotype, N (%)	48 (20.1%)	9 (12.2%)	14 (18.0%)	25 (28.7%)	0.029
Median frequency of MT allele among WT, % (range)	1.7 (0–39.8)	2.0 (0–32.0)	2.2 (0–35.8)	0.8 (0–39.8)	NA
Median frequency of MT allele among MT, % (range)	94.6 (2.0–97.6)	94.1 (2.0–96.2)	92.8 (39.6–94.8)	96.1 (89.0–97.6)	NA
A613S§					NA
Median frequency of MT allele among WT, % (range)	3.9 (0.5–10.7)	3.9 (0.5–7.3)	5.3 (1.3–10.7)	3.3 (0.9–6.3)	NA
Median frequency of MT allele among MT, % (range)	3.4 (1.6–97.5)	4.6 (2.9–97.5)	5.5 (3.5–13.1)	2.8 (1.6–6.0)	NA
Median frequency of any mutation among WT, % (range)	6.5 (0.5–43.2)	6.4 (0.5–34.6)	8.4 (1.3–41.8)	4.7 (0.9–43.2)	NA
Median frequency of any mutation among MT, % (range)	98.8 (52.7–99.7)	98.9 (70.3–99.6)	98.7 (52.7–99.4)	98.8 (94.2–99.7)	NA
A437G¶					
Mutant genotype, N (%)	214 (88.8%)	66 (88.0%)	68 (86.1%)	80 (92.0%)	0.473
Median frequency of MT allele among WT, % (range)	0.4 (0–49.0)	0.4 (0–36.8)	1.6 (0–49.0)	0 (0–46.4)	NA
Median frequency of MT allele among MT, % (range)	93.3 (50.3–97.9)	95.3 (52.7–97.7)	94.9 (52.9–97.9)	89.2 (50.3–97.3)	NA
S436A**					
Mutant genotype, N (%)	104 (43.3%)	26 (36.7%)	33 (43.4%)	45 (51.7%)	0.167

dhps resistance mutations	All years (N = 243)	Time period 2008–2009 (N = 75)	2013–2014 (N = 79)	2017–2018 (N = 89)	P value
Median frequency of MT allele among WT, % (range)	1.3 (0–41.5)	1.1 (0–41.5)	1.7 (0.3–39.1)	1.3 (0.5–37.0)	NA
Median frequency of MT allele among MT, % (range)	97.2 (50.9–99.0)	97.7 (50.9–99.0)	97.2 (52.9–98.8)	96.9 (61.2–98.8)	NA
S436F††					
Mutant genotype, N (%)	3 (2.6%)	1 (2.8%)	2 (5.0%)	0 (0%)	0.411
Median frequency of MT allele among WT, % (range)	0.3 (0–41.5)	0.4 (0–41.5)	0.4 (0–2.9)	0 (0–2.5)	NA
Median frequency of MT allele among MT, % (range)	97.1 (96.4–97.5)	97.5 (97.5–97.5)	96.7 (96.4–97.1)	NA	NA

*MT, mitochondrial; NA, not applicable WT, wild type
†Missing: 1 (2013–2014)
‡Missing: 1 (2008–2009); 4 (2013–2014); 17 (2018–2019)
§Missing: 1 (2008–2009); 1 (2013–2014); 2 (2018–2019)
¶Missing: 2 (2017–2018)
**Missing: 4 (2008–2009); 3 (2013–2014); 2 (2017–2018)
‡‡Missing: 39 (2008–2009); 39 (2013–2014); 48 (2017–2018)

Appendix Table 4. Resistance mutations and copy number for *mdr1**

mdr1 resistance mutations	All years (N = 243)	Time period 2008–2009 (N = 75)	2013–2014 (N = 79)	2017–2018 (N = 89)	P value
N86Y†					
Mutant genotype, N (%)	49 (21.1%)	32 (42.7%)	11 (14.3%)	6 (7.5%)	<0.001
Median frequency of MT allele among WT, % (range)	2.5 (0.6–45.6)	2.0 (0.8–39.7)	3.0 (0.6–45.6)	2.5 (0.9–42.2)	NA
Median frequency of MT allele among MT, % (range)	100 (50.4–100)	100 (60.6–100)	100 (50.4–100)	95.8 (89.3–100)	NA
Y184F‡					
Mutant genotype, N (%)	128 (54.2%)	37 (49.3%)	47 (60.3%)	44 (53.0%)	0.396
Median frequency of MT allele among WT, % (range)	1.6 (0–45.9)	1.3 (0–36.4)	1.7 (0–45.9)	1.7 (0–44.7)	NA
Median frequency of MT allele among MT, % (range)	92.1 (51.4–97.6)	92.8 (56.0–96.7)	89.1 (51.4–97.6)	91.4 (74.4–97.3)	NA
N1042D§					
Mutant genotype, N (%)	0 (0%)	0 (0%)	0 (0%)	0 (0%)	1.0
Median frequency of MT allele among WT, % (range)	5.2 (0–31.7)	4.3 (0–31.7)	5.4 (3.0–9.6)	6.5 (3.3–15.7)	NA
D1246Y¶					
Mutant genotype, N (%)	19 (7.9%)	13 (17.6%)	3 (3.8%)	3 (3.5%)	0.003
Median frequency of MT allele among WT, % (range)	9.8 (2.5–34.9)	7.4 (4.4–15.9)	8.2 (2.5–34.9)	16.3 (5.0–27.6)	NA
Median frequency of MT allele among MT, % (range)	93.1 (51.3–98.2)	93.1 (51.3–97.5)	84.1 (59.6–96.6)	97.1 (61.3–98.2)	NA
S1034T**					
Mutant genotype, N (%)	1 (0.43%)	0 (0%)	0 (0%)	1 (1.2%)	1.000
Median frequency of MT allele among WT, % (range)	1.0 (0–46.8)	0.9 (0–27.9)	0.8 (0.4–2.1)	1.4 (0.8–46.8)	NA
Median frequency of MT allele among MT, % (range)	95.9 (95.9–95.9)	NA	NA	95.9 (95.9–95.9)	NA
S1034R††					
Mutant genotype, N (%)	0 (0%)	0 (0%)	0 (0%)	0 (0%)	1.000
Median frequency of A mutation among WT, % (range)	0.6 (0–3.5)	0.3 (0–1.5)	0.6 (0–3.5)	0.8 (0–1.9)	NA
Median frequency of G mutation among WT, % (range)	0 (0–2.9)	0 (0–1.8)	0 (0–1.9)	0 (0–2.9)	NA
Median frequency of any mutation among WT, % (range)	0.7 (0–4.7)	0.5 (0–2.2)	0.7 (0–3.5)	1.1 (0–4.7)	NA
pfmdr1 copy number**	1.2 (0.3–5.4)	1.1 (0.8–1.4)	1.1 (0.3–2.0)	1.9 (0.7–5.4)	<0.001

* MT, mitochondrial; NA, not applicable WT, wild type
†Missing: 2 (2013–2014); 9 (2017–2018)
‡Missing: 1 (2013–2014); 6 (2017–2018)
§Missing: 1 (2008–2009); 3 (2017–2018)
¶Missing: 1 (2008–2009); 2 (2017–2018)
**Missing: 3 (2008–2009); 4 (2013–2014); 4 (2017–2018)
‡‡Missing: 2 (2013–2014)

Appendix Table 5. Resistance mutations across *atpase6**

atpase 6 resistance mutations	All years (N = 243)	Time period 2008–2009 (N = 75)	2013–2014 (N = 79)	2017–2018 (N = 89)	P value
atpase 6 A623E†					
Mutant genotype, N (%)	2 (0.85)	1 (1.3%)	1 (1.3%)	0 (0%)	0.548
Median frequency of MT allele among WT, % (range)	0 (0–28.6)	0 (0–13.1)	0 (0–28.6)	3.9 (0–18.71)	NA
Median frequency of MT allele among MT, % (range)	64.2 (62.1–66.3)	66.3 (66.3–66.3)	62.1 (62.1–62.1)	NA	NA
atpase 6 S769N‡					
Mutant genotype, N (%)	0 (0%)	0 (0%)	0 (0%)	0 (0%)	1.000
Median frequency of MT allele among WT, % (range)	4.9 (0–27.4)	4.9 (1.6–10.9)	4.4 (0–8.2)	6.0 (1.4–27.4)	–

* MT, mitochondrial; NA, not applicable WT, wild type
†Missing: 9 (2017–2018)
‡Missing: 1 (2013–2014), 62 (2017–2018)

Appendix Table 6. Resistance mutations across *cytb**

cytb resistance mutations	All years (N = 243)	Time period 2008–2009 (N = 75)	2013–2014 (N = 79)	2017–2018 (N = 89)	P value
cytb Y268N†					
Mutant genotype, N (%)	0 (0%)	0 (0%)	0 (0%)	0 (0%)	1.0
Median frequency of MT allele among WT, % (range)	0 (0–19.0)	0 (0–2.4)	0 (0–8.4)	3.0 (0–19.0)	NA
cytb Y268S‡					
Mutant genotype, N (%)	0 (0%)	0 (0%)	0 (0%)	0 (0%)	1.0
Median frequency of MT allele among WT, % (range)	1.0 (0–8.5)	1.7 (0–5.0)	1.1 (0.7–2.6)	0.9 (0.37–8.5)	NA
cytb Y268C§					
Mutant genotype, N (%)	0 (0%)	0 (0%)	0 (0%)	0 (0%)	1.0
Median frequency of MT allele among WT, % (range)	0.6 (0–18.8)	0.7 (0–1.5)	0.6 (0–18.8)	0.6 (0–3.7)	NA
Median frequency of any mutation, % (range)	1.7 (0–19.5)	2.6 (0–5.6)	1.7 (0.9–19.5)	1.5 (0.5–12.2)	NA

*MT, mitochondrial; NA, not applicable WT, wild type
†Missing: 4 (2017–2018)
‡Missing: 8 (2017–2018)
§Missing: 8 (2017–2018)
